# Effect of *Pleurotus ostreatus* powder addition in vegetable soup on ß‐glucan content, sensory perception, and acceptability

**DOI:** 10.1002/fsn3.917

**Published:** 2019-01-28

**Authors:** Cristina Proserpio, Vera Lavelli, Monica Laureati, Ella Pagliarini

**Affiliations:** ^1^ Department of Food, Environmental and Nutritional Sciences (DeFENS) University of Milan Milan Italy

**Keywords:** check‐all‐that‐apply, fiber, liking, mushroom, sensory perception

## Abstract

*Pleurotus ostreatus* is an edible mushroom with interesting nutritional properties, which is able to grow on agri‐food waste and could in turn be used as an ingredient for food fortification. However, new food products have to face with the growing consumer consciousness about what they eat and hedonic responses, which represent a key factor in determining food preference and choices. The aim of this study was to design a vegetable‐based product (a pumpkin and carrot soup) added with increasing concentration of *P. ostreatus* powder rich in β‐glucans, which are fibers with demonstrated bioactivity in humans, and to obtain a sensory description of these fortified products to find the desirable and undesirable sensory properties that affect their acceptance. A total of 109 subjects (women *N* = 52; men *N* = 57; age = 36.1 ± 14.4 years) evaluated five samples of pumpkin and carrot soup added with increasing concentrations of mushroom powder (0%, 2%, 4%, 6%, and a hidden control at 0%) for liking and sensory properties by means of the check‐all‐that‐apply method. Results showed that creaminess, orange color, mild odor, and taste were positively related to vegetable soups liking, whereas strong taste, dark color, and mushroom odor described the less liked samples. Sample added with 2% of mushroom powder obtained comparable liking scores to the unmodified sample, while liking decreased with increasing concentration of *P*. *ostreatus* powder. The present results demonstrated that it is possible to fortify a vegetable soup with *P. ostreatus* powder developing well‐accepted foods by consumers. This product could be used to implement an everyday dietary intervention of β‐glucans over a long‐term period.

## 1 INTRODUCTION

One of the leading priorities for researchers in today's food industry is to develop sustainable new food formulations with healthy properties. Indeed, with the increasing incidence of cardiovascular disease, type‐2 diabetes, and cancer, there is a need to adopt new dietary strategies and to create foods that could potentially support diseases prevention. Consequently, food industry spends considerable resources in the development of new enriched food products and technologies for designing these foods (Siegrist, Shi, Giusto, & Hartmann, 2015).

The role that medicinal mushrooms can play on human health has been investigated since long times and continues to attract attention (Jayakumar, Thomas, Sheu, & Geraldine, 2011; Lavelli, Proserpio, Gallotti, Laureati, & Pagliarini, 2018). Various biological activities of these mushrooms have been associated with β‐glucans which are present in the cell wall of fungi (Dalonso, Goldman, & Gern, 2015). β‐glucan mushroom consists mainly of linear β‐(1→3)‐linked backbones with β‐(1→6)‐linked side chains of varying distribution and length. These β‐glucans can shape complex tertiary structures which are stabilized by interchain hydrogen bonds (Brown & Gordon, 2005).

*Pleurotus* genus is recognized as one of the most important sources of β‐glucans, particularly pleuran, which has demonstrated bioactivity in humans and it is currently marketed as Imunoglukan P4H1, a natural immunostimulant (Bergendiova, Tibenska, & Majtan, 2011; Bobovčák, Kuniaková, Gabriž, & Majtan, 2010; Jesenak et al., 2013).

Previous studies have investigated the effects of dietary supplementation with *Pleurotus* spp. powder on both healthy and unhealthy humans. Daily doses of 3–5 g of *P. ostreatus* powder were found to decrease fasting plasma glucose level in both healthy people and type‐2 diabetic patients (Agrawal et al., 2010; Choudhury et al., 2013). Slightly higher daily doses, in the range 5–10 g, were found to enhance the innate and acquired immune responses in healthy human subjects (Sun et al., 2014). With daily doses in the range 10–30 g, a decrease in triglycerides, oxidized low‐density lipoprotein, and total cholesterol levels was observed in both healthy humans and patients with dyslipidemia (Kajaba, Simoncic, Frecerova, & Belay, 2008; Schneider et al., 2011). The molecules involved in the health effects of *Pleurotus* spp. and their mechanism of action have not been completely clarified in most cases. However, the major role of β‐glucans of *Pleurotus* spp. (pleuran) in the immunomodulatory properties is well documented and recognized. The mechanism of its action in the organism is mediated through several receptors, especially the dectin‐1 receptors, Toll‐like receptors, complement receptor 3, scavenger receptor, and lactosylceramide. After the binding of β‐glucan to its receptors, it stimulates the production of many cytokines and other mechanisms of immune and nonimmune reactions (Bobovčák et al., 2010). A daily dose of 100 mg of pure pleuran was found to have immunomodulatory properties in subjects with a depressed immunosystem, such as children with respiratory diseases (Jesenak et al., 2013) and athletes (Bergendiova et al., 2011; Bobovčák et al., 2010).

The above‐reported studies suggest that *P. ostreatus* could play a major role in the development of functional foods. Moreover, considering that this mushroom can also grow efficiently on low‐cost substrate (i.e., wood and lignocellulosic agri‐food by‐products), it could play a major role in the development of “sustainable foods” (Ghorai et al., 2009; Lavelli et al., 2018). Various factors such as the increased life expectancy, the impact of the agro‐food system on the environment, and the increasing costs of health care are likely to contribute to the upcoming growth in sustainable foods product segment (Siegrist et al., 2015). However, new food products have to face with the growing consumer consciousness about what they eat and hedonic responses, which represent a key factor in determining food preference and choices (Hayes, Feeney, & Allen, 2013). Few studies have been performed investigating the role of *Pleurotus* spp. as a new food ingredient and how this ingredient could affect sensory perception and acceptability of new food formulations.

The objectives of this study were as follows: (a) to design a vegetable‐based product (a pumpkin and carrot soup) added with increasing concentration of *P. ostreatus* powder rich in β‐glucans; (b) to obtain a sensory description of these fortified products to find the desirable and undesirable sensory properties that affect their acceptance.

A vegetable base product was chosen as model system for the *P. ostreatus* powder addition in order to develop a low energy‐dense product rich in β‐glucans. Low energy‐dense products are requested on the market due to the increasing phenomenon of overweight and obesity. In particular, a pumpkin–carrot soup was chosen based on preliminary tasting sessions, which revealed that this flavor fitted better than other vegetable flavors with the mushroom powder addition.

## 2 MATERIALS AND METHODS

### 2.1 Participants

A total of 109 subjects (mean age: 36.1 ± 14.4 years), 52 women and 57 men, were recruited among students and employees of the Faculty of Agriculture and Food Sciences of the University of Milan. Only subjects being ≥18 years of age, who liked and usually consumed pumpkin–carrot soup and mushrooms, were involved in the study. Every subject was asked for informed consent before the assessments were made.

### 2.2 Stimuli

Widely used commercial strains of *P. ostreatus* were purchased from Società Agricola IoBoscoVivo srl (Vergiate, Varese, Italy) in dried form. Mushroom samples included one batch of the KCS50152 strain (PO1 KC50152) produced in September 2017 and three batches of KCS0160 strain (PO1KCS0160, PO2 KCS0160, PO3 KCS0160) produced in September 2017, December 2017, and May 2018. Mushroom samples were sliced, air‐dried at 40°C, and then ground to a fine powder using a Thermomix TM 31 (Vorwerk Contempora S.r.l., Italy) before determination of total glucans and β‐glucan contents and sensory assessment.

Samples for the sensory evaluation consisted of five different formulations of a pumpkin–carrot frozen soup (Carrefour), which requires only to be heated before consumption. The ingredients of the soup were as follows: pumpkin 47%, water, carrots 17%, onion, salt, garlic, yeast extract, and rosemary (48 kcal per 100 g). The experimental samples were prepared by adding different increasing concentrations (C0 = 0%, C2 = 2%, C4 = 4%, and C6 = 6%) of *P. ostreatus* powder to this standard formulation. A hidden control of the unmodified sample (HC = 0%) was also included. In samples C4 and C6, it was necessary to add water, respectively, 6.7% and 10%, in order to make the samples similar regarding appearance to the others samples.

In order to define the concentrations of mushroom powder to be added to the vegetable soups, pilot triangle tests (Lawless & Heymann, 2010; Proserpio et al., 2016) were performed with a separate group of 20 adults (data not shown). All stimuli were prepared on the same day of the session and were presented at warm temperature. Approximately 30 g of each sample was presented to the participants in plastic cups labeled with three‐digit codes. Water was available for rinsing the palate.

### 2.3 Determination of β‐glucan content

The content of total glucans and β‐glucan was determined in triplicate using the Megazyme assay kit (K‐YBGL, Megazyme International Ireland Ltd, Wicklow, Ireland), following the H_2_SO_4_ acid hydrolysis procedure by McCleary & Draga, 2016.

### 2.4 Experimental procedure for the sensory evaluation

All subjects were invited to the sensory laboratory that was designed according to ISO guidelines (ISO 8589 2007). They were asked to refrain from consuming anything but water for 2 hr before the test. For each sample, subjects had to score their overall liking and to answer a check‐all‐that‐apply (CATA) question. The entire session took approximately 30 min. Data were collected using the Fizz v2.47 software program (Biosystemes).

### 2.5 Liking assessment

Subjects were asked to taste the products and to express their liking scores using a labeled hedonic scale (LAM), anchored by the extremes “greatest imaginable dislike” (rated 0) and “greatest imaginable like” (rated 100) (Schutz & Cardello, 2001). The experimenters provided instructions for the use of the scale prior to tasting, and during the sessions, the instructions were “You will use your mouse to click anywhere on the line to indicate how much you like or dislike the product” (Lawless, Popper, & Kroll, 2010).

### 2.6 Generation of descriptive terms for the CATA assessment

A separate group of 12 untrained subjects aged 20–40 years took part in a pilot test, wherein judges used a free listing questionnaire to establish the appropriate terms to describe the samples (Ares, Deliza, Barreiro, Giménez, & Gámbaro, 2010). They were asked to pay attention to the sensory characteristics of the soups samples and to provide all terms for describing their color, appearance, odor, taste, flavor, and texture. An open discussion followed the development of lexicon. Then, the experimenters finalized the list of terms, selecting the most mentioned (terms reported at by at least 20% of subjects) and the most common word in order to avoid synonymous (Jaeger et al., 2015).

### 2.7 Check‐all‐that‐apply (CATA) assessment

The CATA questionnaire consisted of a list of 24 sensory attributes, including appearance, odor, taste, flavor, and texture terms. Accordingly, it has been suggested that an appropriate list of CATA questions should be comprised of 10–40 terms to taking into account consumers’ heterogeneity but in the meantime avoiding a dilution effect of the responses (Ares & Jaeger, 2015; Jaeger et al., 2015).

The terms considered were the following: 4 for the appearance (orange color, homogeneous, irregular, and dark color), 6 for the odor (pumpkin, carrot, mushroom, mild, strong, and pungent), 4 for the taste (sweet, bitter, sour, and salty), 5 for the flavor (pumpkin, carrot, mushroom, mild, and strong), 2 for tactile sensations (spicy and astringent), and 3 for the texture (floury, grainy, and creamy). Subjects were asked to check from the list all the terms that they considered appropriate to describe each of the samples. The position of attributes was randomized using the “to assessor” list order allocation scheme (Meyners & Castura, 2016).

### 2.8 Data analysis

The contents of total glucans and β‐glucans of mushroom samples were analyzed using one‐way ANOVA with the least significant difference (LSD) as a multiple range test using Statgraphics 5.1 (STCC Inc.; Rockville, MD).

A linear mixed model procedure was carried out on overall liking data considering “samples” (C0, HC, C2, C4, and C6), “gender” (women and men), and their two‐way interaction (“sample” * “gender”) as fixed factors. Age was added to the model as covariate. Participants were considered as random factor in all the analyses. When a significant difference (*p* < 0.05) was found, the LSD *post hoc* test was performed as multiple comparison test.

For the CATA question, the frequency of mention for each term was determined by counting the number of consumers that used that term to describe each sample. Since C0 and HC obtained comparable liking scores in the subsequent analysis, only sample C0 was considered. Cochran's *Q* test was carried out for each of the 24 terms to detect differences in consumers’ perception of the evaluated samples.

Correspondence analysis (CA) was used to get a bidimensional representation of the samples and the relationship between samples and terms from the CATA question (Ares, Dauber, Fernández, Giménez, & Varela, 2014). CA was performed on the frequency table containing responses to the CATA questions, considering the average liking scores by product as supplementary variable. These statistical analyses were performed using XLSTAT‐Sensory^®^ software for Windows, Version 2015.6.01 (Addinsoft^™^, France). A *p*‐value of <0.05 was considered significant.

## 3 RESULTS

### 3.1 β‐glucan content and product design

As shown in Table 1, β‐glucans were the major components of total glucans for both the *Pleurotus* strains. These compounds ranged between 24.9 and 36.9 g/100 g d.w. The three batches of KCS0160 strain obtained in three different months (September, December, and May) showed the same β‐glucan content, which was higher that of the KCS50152. Hence, the KCS0160 was selected for addition to a vegetable soup.

**Table 1:** Content in total glucans (mean ± *SD*, g/100 g d.w.) and β‐glucans (mean ± *SD* g/100 g d.w.) in *P. ostreatus*

Samples	Total glucan	β‐glucan
PO1 KCS0152	25.4^b^ ± 2.3	24.9^b^ ± 2.3
PO2 KCS0160	37.1^a^ ± 2.8	35.0^a^ ± 2.7
PO3 KCS0160	39.1^a^ ± 2.5	36.9^a^ ± 2.5
PO4 KCS0160	36.0^a^ ± 0.9	35.0^a^ ± 0.9

^a,b^Different letters in the same column represent significant differences (LSD, *p *<* *0.05).

Few studies have been carried out investigating how the addition of mushroom powder could affect the ß‐glucan daily intake (Table 2). In the present study, *P. ostreatus* powder was added at levels 6, 12, and 18 g of dried mushroom per single portion of soup (300 g). Hence, a single portion of the fortified soups could provide 2.2, 4.4, and 6.6 g of ß‐glucans, respectively. Based on the in vivo studies above described in the introduction, the amount of *Pleurotus* powder added is in the range of the intake that is likely to have positive health effects (3–30 g daily).

**Table 2:** Functional foods enriched with mushroom β‐glucans

Food	Ingredient and addition level (%)	β‐glucan per serving (g)^a^	References
Rice noodles	*L. edodes* insolule β‐glucan‐ fraction 4, 8, 12	1.1–3.4 (100)	Heo et al. (2014)
Baked cake	*L. edodes* insolule β‐glucan‐ fraction 1, 2, 3	1–3 (100)	Kim et al. (2011)
Pasta	*P. eryngii* insoluble β‐glucan‐ fraction 2, 4, 6	0.79–4.8 (100)	Kim et al. (2016)
Biscuits	*P. sajor‐caju* powder 4, 8, 12	0.32‐0.53 (30)	Ng et al. (2017)
Vegetable soup	*P. ostreatus* powder 2, 4, 6	2.2–6.6 (300)	This study

a Values in brackets indicate the portion size.

### 3.2 Liking assessment

**Figure 1:**
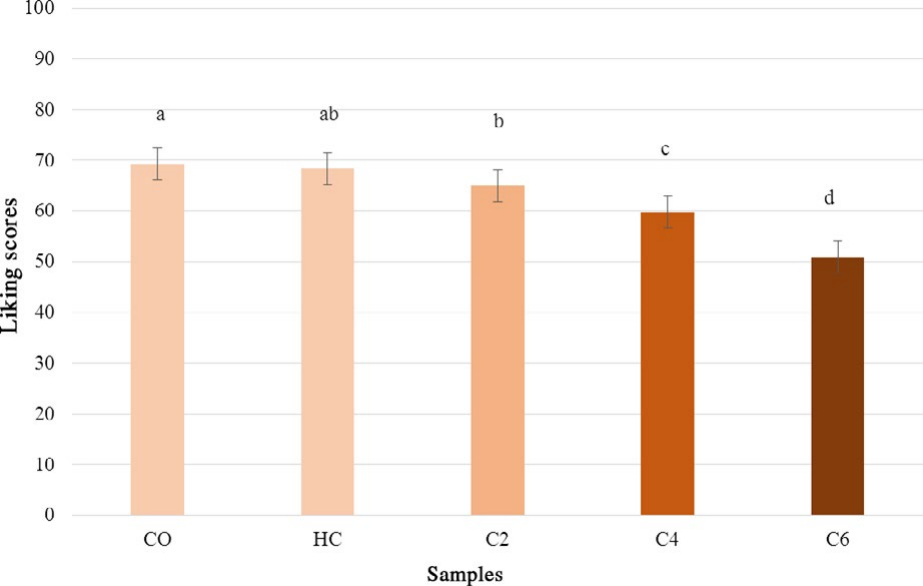
Mean liking scores ± *SEM* by samples. Different letters indicate significant differences according to *post hoc* test

The mean liking scores by samples are provided in Figure 1. The main factor “samples” was found to have a significant effect on liking (*F*_(4,428)_ = 46.67, *p* < 0.001). C2 sample obtained liking scores comparable to the unmodified sample HC, which was in turn comparable to C0. The addition of increasing concentrations of *P. ostreatus* powder produced a decrease in liking for sample C4 and C6 even if the scores are still up the middle of the scale (score 50) corresponding to an acceptable product. The main factor “gender” and the interaction “sample” * “gender” were not significant (*F*_(1,107)_ = 1.38, *p* = 0.24; *F*_(4,428)_ = 0.54, *p* = 0.70, respectively).

### 3.3 CATA assessment

The frequency table of terms checked by consumers to describe the five samples is reported in Table 3.

**Table 3:** Frequency mention of sensory attributes associated with each samples

Sensory modality	Sensory attributes	Frequency of mentions			
		Samples			
		C0	C2	C4	C6
Appearance	Orange color***	97	51	23	8
	Homogeneous***	80	39	23	10
	Irregular***	4	44	51	68
	Dark color***	7	42	65	70
Odor	Pumpkin***	56	28	28	18
	Carrot***	38	24	16	9
	Mushroom***	7	46	54	76
	Mild***	68	50	40	20
	Strong**	8	19	21	29
	Pungent**	1	10	9	14
Taste	Sweet***	72	46	35	21
	Bitter***	0	3	15	38
	Sour***	6	2	6	18
	Salty n.s.	12	19	20	11
Flavor	Pumpkin***	63	43	44	26
	Carrot***	63	41	33	23
	Mushroom***	8	46	56	82
	Mild***	66	44	29	15
	Strong***	8	22	39	49
Tactile	Spicy n.s.	6	8	8	14
	Astringent**	7	5	10	20
Texture	Floury***	6	25	24	31
	Grainy***	7	35	59	74
	Creamy***	96	56	47	29

*Notes*

n.s., nonsignificant difference according to Cochran's *Q* test.

Significant difference for *^*^ *p* < 0.01; *^**^ *p* < 0.001.

As shown in Table 3, significant differences were found in the frequency mention for 22 out of 24 terms within the six categories considered, suggesting that consumers perceived differences between samples in terms of their sensory characteristics. The sensory attributes that were not useful in order to discriminate samples were “salty” and “spicy.” Indeed, looking at the frequency of mention of these attributes, these terms were used quite homogeneously and were checked by less than half of the respondents, indicating that the consumers’ consensus was low.

### 3.4 Relating sensory profiling (CATA) with liking

**Figure 2:**
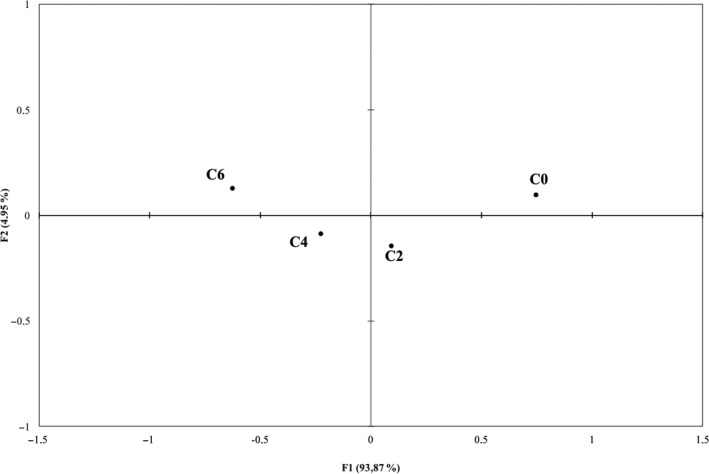
Products plot obtained from CATA

The CA performed on the total frequency counts for each attribute resulted in two dimensions accounting for 98.82% of the variance in the data. As inferred from Figure 2, samples were discriminated according to mushroom powder addition. C0 sample was positioned in the upper right side of the map while samples added with 4% and 6% of powder were positioned in the left side of the map. Sample C2 was positioned near to the C4 sample but in the right side of the map.

**Figure 3:**
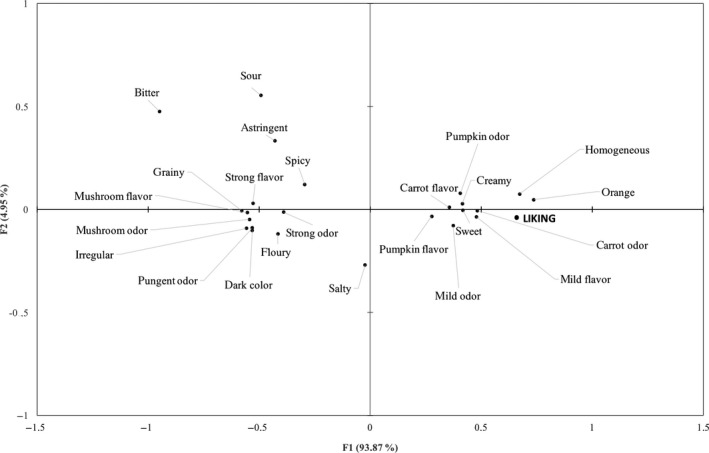
Attributes plot obtained from CATA total frequency counts and liking

The relation between sensory terms and overall liking of the four samples is reported in Figure 3.

Comparing Figures 2 and 3, it is possible to see that subjects’ liking was oriented toward the sample without the addition of the powder and C2 on the right side of the map. Liking scores were mainly associated with the sensory descriptors “creamy,” “orange color,” “mild flavor,” “mild odor,” “sweet,” “pumpkin odor,” and “carrot odor.” C6 sample with the highest amount of *P. ostreatus* powder, which obtained the lowest liking scores, was mainly described by the terms: “strong taste,” “mushroom odor,” “grainy,” and “dark color.”

## 4 DISCUSSION

In the present study, vegetable soups with increasing amounts of β‐glucans from *P. ostreatus* powder were designed. A sensory description of these products was performed to find the desirable and undesirable sensory properties that affect consumers’ acceptance. To the best of our knowledge, this is one of the first studies that examined how mushroom powder addiction could affect sensory properties and hedonic responses applying validated sensory methods.

Due to the increasing consumers’ interest in health, food industries are focused on the development of new food products with improved functionality (Siegrist et al., 2015). However, these new food products need to be accepted by the consumers as part of their daily diet and, therefore, a better understanding of how consumers perceive these formulations is needed. It is well known that functional benefits may provide added value to consumers; however, these benefits do not compensate consumers’ perception of sensory properties, which is the main driver of food choices (Hayes et al., 2013). Indeed, consumers base their choices more on pleasantness than perceived healthiness. Thus, the use of fiber‐rich ingredient in developing new fortified foods needs to be evaluated not only from an analytical point of view, but also by investigating consumers’ perception.

The present results demonstrated that it is possible to fortify a vegetable base product with *P. ostreatus* powder developing well‐accepted samples by consumers. Indeed, liking of sample added with low concentration of mushroom was comparable to the unmodified sample. Moreover, even if the liking decreased with increasing concentration of mushroom powder, sample added with 4% and 6% was still acceptable by consumers, since mean liking scores were higher than half of the hedonic scale (score 50). Moreover, from the CATA analysis, the evaluated samples were significantly discriminated among them in terms of sensory descriptors, thus the addition of the mushroom powder significantly changed consumers’ perception. The decrease in liking ratings could be due to changes in sensory characteristics related to appearance, which has a great influence on consumers’ choice, and a pronounced mushroom taste and flavor.

Despite the demonstrated health benefit of *Pleurotus* β‐glucans, few studies have focused on the development of foods enriched in these compounds. These latter studies include applications of β‐glucans from two basidiomycetes, that is, *Lentinus* and *Pleurotus*. One approach consisted in the isolation of the insoluble β‐glucan‐rich fraction before addition to a target food. Heo, Jeon, and Lee (2014) developed rice noodles with 4.8 g of the insoluble β‐glucans from *L. edodes* per serving (100 g). However, the sensory evaluation was not performed on these samples. Kim et al. (2011) have proposed the addition of insoluble β‐glucans from *L. edodes* into a baked cake and found that the formulation containing 1 g of β‐glucan per serving (100 g) resulted in similar volume and textural properties to the control. Nevertheless, a sensory evaluation was not performed on the developed samples.

Similar to the present research, previous studies investigated how the addition of *Pleurotus* spp. powder could affect the sensory properties of some cereal‐based products (e.g., bread, biscuits, and pasta) but no studies have been performed trying to design a low energy‐dense vegetable base product, which are always more requested on the market due to the increasing phenomenon of obesity (Proserpio et al., 2016). Recently, Kim, Lee, Heo, and Moon (2016) have proposed the application of the insoluble β‐glucan fraction separated from mushroom powder in pasta. The results of sensory evaluation showed that common wheat pasta obtained the lowest liking scores, while the acceptability increased with the addition of the insoluble β‐glucan fraction. In particular, the sample with 2% of the β‐glucan‐rich fractions added to replace wheat flour was significantly preferred compared to the sample without supplementation. However, for the preferred formulation, the amount provided per serving was only 0.79 g. Ng, Robert, Ahmad, and Ishak (2017) found that supplementation with *P. sajor‐caju* powder up to 8% to biscuits could lead to a more desirable aroma, color, and flavor when compared with the biscuit without supplementation. Nevertheless, with higher amounts of *P. sajor‐caju* powder, undesirable results were obtained, with decreasing liking scores due to the higher degree of firmness and the stronger aroma and flavor as well as the darker surface color of the biscuits. For this fortified food, the amount delivered was 0.38 g per serving.

Based on the above‐mentioned studies, it can be confirmed that the vegetable soup developed in the present study can be a good matrix for β‐glucan incorporation. According to the in vivo studies so far performed, the amount added to the sample which obtained the highest liking scores is in a range that has potential to provide health benefits. In fact, being a low energy‐dense food, the vegetable soup could be provided in a large size.

## 5 CONCLUSIONS

The results of the present study suggest that it is possible to develop new β‐glucans enriched products using an adequate concentration of mushroom powder without decreasing consumers’ satisfaction. These fortified foods could be considered more beneficial to improving health than the conventional soups. Indeed, vegetable soup is already rich in fiber but mushroom β‐glucans could improve product functionality. The low energy‐dense vegetable soup designed in this study can be assumed in a large serving size and contribute to a significant intake of mushroom β‐glucans to implement an everyday dietary intervention over a long‐term period. Thus, new food formulations could be developed using ingredient obtained from low‐cost resources able to grow on substrates obtained from by‐products and wastes of the food chain.

## AUTHOR CONTRIBUTIONS

The author contributions were as follows: CP, VL, and EP designed the study. CP carried out the experiment, performed the statistical analysis, and wrote the manuscript. CP, VL, ML, and EP regularly discussed the experiment, analyzed the results, and provided useful suggestion during the writing. All authors read and approved the final manuscript.

## ETHICAL STATEMENT

The authors declare no conflict of interest. The present study was performed according to the principles established by the Declaration of Helsinki, after the protocol was approved by the Ethical Committee (University of Milan).
